# Fascia Tissue Manipulations in Chronic Low Back Pain: A Pragmatic Comparative Randomized Clinical Trial of the 4xT Method^®^ and Exercise Therapy

**DOI:** 10.3390/life14010007

**Published:** 2023-12-20

**Authors:** Robbert van Amstel, Karl Noten, Shaun Malone, Peter Vaes

**Affiliations:** 1 Department of Human Movement Sciences, Faculty of Behavioural and Movement Sciences, Amsterdam Movement Sciences, Vrije Universiteit Amsterdam, 1081 Amsterdam, The Netherlands; 2 Fysio Science Department, Fysio Physics Groups, 3401 IJsselstein, The Netherlands; 3 Department of Rehabilitation Sciences and Physiotherapy (MOVANT), University of Antwerp, Wilrijk, 2000 Antwerpen, Belgium; 4 Faculty of Rehabilitation Science and Physical Therapy, Vrije Universiteit Brussel, 1050 Brussels, Belgium; pvaes@vub.be

**Keywords:** myofascial release therapy, athletic tape, exercise program, physical therapy modalities, low back pain, range of motion, articular, quality of life

## Abstract

Background: The 4xT method is a protocolized practice in treating musculoskeletal disorders. The 4xT method consists of four components: Test (functional diagnostic test), Trigger (fascia tissue manipulations), Tape (elastic taping), and Train (exercise). There is a lack of clinical studies evaluating the treatment effects of the use of the 4xT method. Methods: A randomized controlled trial was conducted to compare the effectiveness of the 4xT method and exercise therapy-only in patients with chronic nonspecific low back pain. Based on a priori sample size calculation, fifty-one individuals with chronic nonspecific low back pain were randomly assigned to either the 4xT or exercise group. Both groups underwent a six-week rehabilitation program with two treatments per week. The primary outcomes were trunk flexion and extension mobility, trunk flexion, and extension mobility-dependent pain, and quality of life evaluated during a 6-week therapy period and after a 6-week therapy-off period. Results: Interaction effects were noted in all outcomes. The 4xT group showed significant improvements over time for trunk flexion and extension mobility, trunk flexion and extension mobility-dependent pain, and quality of life (*p* < 0.05), with no significant relapse post-therapy (except for extension mobility). The exercise group exhibited significant within-time changes in the quality of life, as measured with the VAS (*p* < 0.05), but not for EQ-5D-3L. Conclusions: The results of this study demonstrate that the 4xT method stands out as a promising and impactful treatment option for chronic nonspecific low back pain individuals, as it demonstrated significant reductions in mobility-dependent pain, increased trunk mobility, and improved quality of life compared to exercise-only treatments.

## 1. Introduction

Low back pain (LBP) is a debilitating condition that affects millions of working individuals worldwide, causing significant suffering and disability [1]. The incidence and prevalence of LBP have been reported to range from 0.02–7.0% and 1.4–20.0%, respectively [2], which is higher in high-income countries than in low-middle-income countries [2,3], and is associated with disability, stress and sickness absence, and reduced labor productivity [4]. The prevalence is expected to increase because of aging, and more LBP in people over the age of 60 years is anticipated [5]. Ninety percent of all LBP patients are classified as nonspecific, and the underlying cause is unknown [6]. While the biopsychosocial factors play a significant role in chronic LBP and the progression from acute to chronic stages [7], it is crucial not to overlook the biomedical elements within the broader biopsychosocial context [8,9]. The most frequently reported symptom among individuals in this population is pain that originates from the thoracolumbar junction and extends to the gluteal area and upper leg [6]. The perception of LBP is subjective and can arise from nerve endings that have become sensitized within the thoracolumbar fascia and lumbar back muscles [10,11,12], which may cause muscle hyperactivity influencing the spine, pelvis, and hip mobility [13,14,15].

The literature and the clinical practice guidelines in the field of physical therapy addressing the management of LBP advocate for a range of interventions [16,17,18,19,20]. This includes a more individualized approach, commonly practiced, and recognized for its potential to be more effective for individuals with LBP [21]. Fascia Tissue Manipulations (FTMs) are novel interventions used by clinicians within an individualized approach. In this paper, we define FTMs as an umbrella term for various interventions targeting the fasciae, with the term ‘fasciae’ being defined by the Fascia Research Group [22,23]. FTMs encompass methods such as soft tissue mobilizations, myofascial release techniques, joint (high-velocity thrust) mobilizations, and the application of elastic tape. They are hypothesized to reduce muscle and fascia stiffness, modulate loose connective tissue, cause the disaggregation of hyaluronic acid, release tissue adhesions, break collagen cross-links, and realign collagen fibers [24,25,26]. Despite these unproven claims, FTMs have been reported to have small to moderate positive effects on pain and disability reduction and function improvement [27,28]. To achieve the maximum effect on reducing pain and improving mobility, it is crucial to identify the optimal location and direction for myofascial release, elastic tape application, and joint mobilization, which are essential components of patient treatment [29]. In response to this challenge, Noten and Schuurman-Stekhoven developed a test procedure that aims to identify the most suitable locations and directions for these interventions [29,30], forming the basis of the 4xT method developed by Noten [30].

The 4xT method is a protocol that claims to select the most appropriate interventions, including location, direction, and intensity, for treating musculoskeletal conditions. The 4xT method consists of four crucial components: Test (functional diagnostic test), Trigger (myofascial release and joint mobilizations), Tape (elastic taping), and Train (exercise) [30]. The method relies on the Dynamic ArthroMyofascial Translation Test^®^ procedure (Test) to guide decision-making. This Test consists of a joint motion with ongoing skin displacement (SKD), allowing therapists to evaluate its effect on pain and range of motion and customize interventions for each patient to achieve maximum pain relief and mobility improvement [30]. A study reported that ongoing lumbodorsal SKD during trunk motion can impact the trunk range of motion in healthy individuals compared to sham SKD [29]. The location and direction of SKD were found to be important factors in the observed instantaneous changes [29]. The study also demonstrated good reliability between different testers and the same tester when conducting the Test [29]. In the 4xT method, the Test, Triggers, and Tape are algorithmically applied at various locations and executed until the patient can move with an acceptable level of pain, allowing them to begin their rehabilitation training [30]. This claim was supported in a case report involving two nonspecific chronic LBP patients [31]. However, there is a lack of controlled clinical studies evaluating the treatment effects of the 4xT method in a larger sample. Only one pilot study reported that treating nonspecific chronic LBP (NSCLBP) individuals according to the 4xT method may be promising in improving the short-term quality of life, increasing trunk mobility, and reducing pain during motion [32]. However, the literature reported that more research is needed to validate the potential benefits of the 4xT method in managing NSCLBP [29,31,32]. The purpose of this study was to evaluate the effectiveness of the 4xT method in NSCLBP individuals by comparing it with exercise-only therapy, using three outcome measures: the change in trunk Range of Motion (ROM), mobility-dependent pain, and quality of life. The effects were measured during a 6-week treatment period; furthermore, it was analyzed how long these effects lasted using a 6-week therapy-off period.

## 2. Materials and Methods

### 2.1. Research Design

The NSCLBP individuals were randomly assigned to a 4xT-group or an exercise therapy group. All tests and measures were conducted at various time points: before allocation to the group (baseline), during the third week of the treatment period (W3), after the 6-week therapy-on period (W6), and following a 6-week therapy-off period (W12). The individuals were treated twice a week according to the program to which they were allocated.

The registered 4xT physiotherapist who treated the patients in the 4xT group [33] completed the comprehensive 4xT Orthopedic Rehabilitation for LBP program, which provided 24 h of practical training and 8 h of e-learning [34]. The physiotherapist gained approximately 600 h of clinical experience with the 4xT method over one year before the study was conducted. The physiotherapist in the exercise therapy group had completed various exercise courses, including motor control, core stability, and medical fitness, and possessed over 3 years of clinical experience in treating individuals with NSCLBP using exercise therapy before the study was conducted.

The study was conducted in accordance with the Declaration of Helsinki, and approved by the Committee of Medical Ethics University Hospital University of Brussels, B.U.N. 143201627110 and it was prospectively registered: (NCT03309540).

### 2.2. Recruitment

The individuals were recruited between October 2017 and December 2019 using an advertisement in a Dutch-language newspaper (Algemeen Dagblad Utrecht, Utrechtse Courant) and social media (Facebook). Moreover, individuals were recruited via direct access consult physiotherapy, via physiotherapy clinics as part of the Fysio Physics groups, the Netherlands. All individuals were informed, read, and signed the informed consent.

### 2.3. Inclusion and Exclusion Criteria

Before taking part in this study, a red flag screening for NSCLBP (SpotOnMedics^®^, Hoofddorp, The Netherlands) of the individuals was carried out by the first author.

Individuals were included when they met the following criteria: NSCLBP for 12 weeks or longer (with and without recurrent complaints), Numeric Rating Scale for Pain ≥ 4 (PNRS; no pain 0–worst pain 10) during the treatment period, age between 25 and 60 years, and unfamiliar with the 4xT method. We have chosen this age category because disability due to low back pain is highest or most severe at the age of 25–65 years [35].

Individuals were excluded when they suffered from: radiating disturbing pain beyond the knee, neurological disorder symptoms, overall malaise, spinal cord malignancy, unexplained weight loss, prolonged corticosteroid use, osteoporotic vertebral fracture, spondylitis ankylopoetica, spinal stenosis, rheumatic arthritis, vertebral fracture, and severe spinal cord deformity [16].

### 2.4. Randomization

The enrolled individuals were randomly assigned to either the 4xT group or the exercise-only group (EGR), with the latter also receiving paravertebral sham elastic tape. The allocation was performed at random using a computer-generated randomized table.

### 2.5. Outcome Measures

In this study, the sagittal trunk ROM was evaluated utilizing the standardized test protocol as described in the literature [32,36] to make sure that each movement was performed comparably. One baseline bubble inclinometer (Model 10,602 built by Fabrication Enterprise Inc., Elmsford, NY, USA) was used. The reliability of repeated measurements was guarded using a reference marker placed between the two spinal processes (Leukoplast Classic, BSN Medical, Hamburg, Germany) for inclinometer placement and was performed by a trained operator to ensure that during the observation the inclinometer position was maintained. The ROM (mean of 3 measurements) was quantified by (1) total trunk extension, (2) total trunk flexion, and (3) lumbar flexion (resp. EROM, FROM, LROM). For total trunk ROM assessments, the inclinometer was placed on L1-T12 (ThoracoLumbar junction) and for LROM on S1-S2 (Sacrum) with the tape between the inclinometer arcus. LROM was calculated by subtracting S1-S2 from T12-L1 [37]. The inclinometer was initially set to 0° while the patient was in a standing position. During trunk motion, the researcher maintained the inclinometer in a stable position, as described in detail in van Amstel et al. [32].

The level of experienced mobility-dependent pain by PNRS was evaluated for the end EROM- and FROM (PNRS_E_; PNRS_F_) [38]. Moreover, the quality of life was measured using the EQ-5D-3L index and the EQ-VAS, developed by Euroqol Group (reg.nr. EQ: ID16666). All tests were evaluated two days after the last treatment without elastic tape in situ.

### 2.6. Blinding

The 4xT physiotherapist, research assistant, and operator (first author) were aware of the interventions used in this study. Patients were blinded to the intervention of the other group, and the exercise therapy group physiotherapist was blinded to the 4xT group intervention. Moreover, the physiotherapist of the exercise therapy group was not informed that the applied paravertebral elastic tape was a sham.

To reduce bias and ensure objective and unbiased data, The ROM data were assessed by an independent assessor who was blinded to the entire study and the operator. Data were recorded separately and concealed by the research assistant. In addition, the PNRS (PNRS_E_; PNRS_F_) and the EQ-5D-3L were evaluated by the same independent assessor.

### 2.7. Exercise Group

The physiotherapist who treated the 4xT-group applied sham tape in the individuals of the EGR, which involved two applications of paravertebral elastic adhesive tape with 0% tension, as previously suggested by other studies [39,40]. After the tape was applied, the back of the patient was covered with clothing. The physiotherapist of the exercise therapy group, who was not informed about the 4xT-group and sham intervention content, conducted the exercise program. This physiotherapist specialized in back rehabilitation training and adjusted the exercise program on the treatment day (twice a week). The exercises consisted of: recumbent bike, abdominal crunch machine, chest press machine, child pose Pilates position, Pilates half rollback, crosstrainer, lower back extension machine, lat pull down, puppy Pilates position, and Pilates-breaststroke exercise (Table S2, Training Program). Under supervision, the individuals performed all the exercises listed in the exercise program.

The rationale behind the training program is to combine flexibility and resistance exercises, as recommended in the LBP guidelines [16,41]. Flexibility training aims to increase functional mobility [42], while resistance training specifically focuses on promoting muscle growth [43] associated with anti-inflammatory responses [44]. The training protocol focuses on isolating the lumbar multifidus and transverse abdominal muscles during muscle strength training, which are considered core muscles and are essential for trunk stability in all directions [45]. To activate the core muscles during Pilates and machine exercises, anisometric–isotonic and anisometric–anisotonic contractions were performed by the patients, respectively. For muscle endurance resistance training, the patients performed 4 sets of 15 to 20 repetitions at a super-slow tempo (8 s for the concentric phase and 8 s for the eccentric phase) at an intensity of 50% 1RM ≤ 1RM. For muscle strength resistance training, the patients performed 4 sets of 8 to 12 repetitions with an eccentric emphasis at an intensity of 70% to 85% of 1RM (1 s for the concentric phase and 4 s for the eccentric phase) [43]. A rest period of 60 s was taken between sets, consistent with the recommendation by Schoenfeld et al. [43].

### 2.8. 4xT Method: Back Protocol

**Figure 1:**
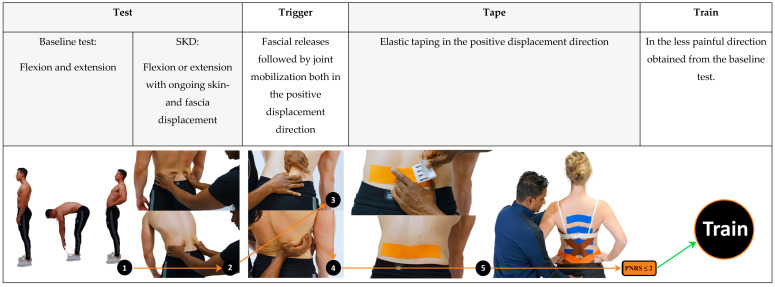
The Dynamic ArthroMyofascial Translation test: a functional diagnostic test procedure. This figure displays the full DAMT test procedure of L3 as an example of obtaining the Trigger and Tape direction. TEST: First, the subject underwent a baseline test (1) to obtain the flexion and extension trunk range of motion and pain intensity at the end position (PNRS). Subsequently, the most painful spinal movement direction (flexion or extension) (highest PNRS score) was used as a reference test to test the effect of displacing the skin and underlying fascia (SKD) on the range of motion and level of pain. The reference test was performed with ongoing mediolateral SKD to the left and right (2). The three tests (reference test including: no SKD or left SKD or right SKD) were compared by the patient in choosing the best condition as the so-called ‘positive’ direction. TRIGGER: The positive direction was the direction of the triggers which started with softening the superficial fascia towards the deeper myofascia (3). After softening the tissues, the tested spinal segment (e.g., L3) was mobilized in the direction of the positive SKD test and in the opposite direction of the reference test, and (4) 3D spinal joint mobilization L3). TAPE: Subsequently, the elastic tape was applied by rubbing the skin and fascia in the mediolateral positive direction. After the full procedure (steps 1–5), the steps were repeated for the next location (e.g., from 1st step L3 to 2nd step S2) until the patient could move with an acceptable level of pain so the patient was able to perform the prescribed training. This flow chart is for clarifying this article. Just studying the images and reading this article does not guarantee successful results, as it requires training according to the 4xT Method^®^. Represented photos are from the 4xT manual with permission of the author Karl Noten [30]).

A registered 4xT physiotherapist trained in the 4xT Back protocol [33] performed the interventions in the 4xT-group. The interventions followed the Test Trigger Tape Train algorithm (Video S1). The intervention in the 4xT-group started with the Dynamic ArthroMyofascial Translation Test, also known as the DAMT-Test procedure (Test) (Figure 1, image 1), to determine the direction and location of the Trigger and Tape. The Test, Trigger, and Tape were sequentially executed at the following locations: first L3, second S2, third T12, fourth mid thoracic spine (T9), fifth high thoracic spine (T4), sixth thoracolumbar fascia/latissimus dorsi right and seventh left, eighth ilium right and ninth left and, finally, tenth step vertical over the lumbar spine (Figure 1, image 5), until the patient was able to move with an acceptable pain level (PNRS ≤ 2), required for rehabilitation exercise (Train). Below is a detailed explanation of the entire procedure, which applies to all locations, using L3 as an example.

**Test:** Each subject started standing straight up. During the baseline test, a maximal trunk flexion and maximal extension movement were performed to retrieve the baseline values (PNRS and ROM) (Figure 1, image 1). The most painful trunk motion (highest Pain NRS score) was selected as the reference test, and the direction causing the least pain was identified as the directional preference (lowest Pain NRS score). The reference test was used to examine the effect of displacing the skin and underlying fascia on both Pain NRS and ROM. The reference test with ongoing SKD was carried out, conform the 4xT Back protocol [30]. The SKD consisted of: (a) a mediolateral directed lumbodorsal SKD to the right and left direction at the height of the spinal locations L3, S2, T12, T9, and T4 (Figure 1, image 2), subsequently; (b) diagonal cranial and caudal SKD above the latissimus dorsi muscle; (c) anterior and posterior SKD above the ilium; and (d) cranial-and caudal SKD over the lumbar spine. The SKD intensity was beyond the skin and underlying fascia slack (grade 4) equivalent to Maitland’s passive tissue stretch grading scale [46,47]. The SKD direction that resulted in the most experienced improvement (increased ROM; decreased PNRS) was chosen as the ‘positive’ and best Trigger and Tape direction at this moment. If the reference test without SKD yielded the most positive outcome for pain and/or trunk ROM, no intervention was applied at that specific location.

**Trigger:** At the targeted location, a cluster of Triggers were applied in a standing position with slight flexion (≈60°) [48] and posterior pelvic tilt, while leaning forward on a physio plinth. The fasciae and muscles were then released using the open fist and adduction technique (Figure 1, image 3). Subsequently, spinal mobilization (grade 4) was performed to mobilize the spinal segment (e.g., L3) in the directional preference and the positive SKD direction (e.g., trunk flexion including right SKD L3 = trunk extension including left L3 rotation mobilization). The medial aspect of the palm was placed at the arcus posterior, and the fifth phalange was positioned approximately at the process transverses L3 while the patient was standing. The physiotherapist assessed the spinal slack in the SKD direction and instructed the patient to take a deep breath, which was then held for 4 s. During exhalation, the joint was mobilized by moving the segment in the SKD direction, which was held for 4 s and repeated four times consecutively (Figure 1, image 4). After the spinal mobilization, the elastic tape was applied in the positive mediolateral SKD direction at L3 while the subject was leaning forward on the physio plinth.

**Tape:** the elastic tape was applied in the positive SKD direction with the Fascia Displacement Technique utilizing ≈20 cm long elastic adhesive tape (EASYTAPE^®^, Msys B.V.) [30]. For example, at L3, the anchor (¼) of the elastic adhesive tape was applied on the skin, ≈7 CM laterally of the spinous process L3. The elastic adhesive tape (¾) was rubbed out with medium-high pressure to the contralateral direction (Figure 1, image 5) [31], to align the tissues underneath the tape [30,49]. Subsequently, the reference test was retested with tape in situ to evaluate the FTMs effect on pain and mobility. Subsequently, the full procedure was executed at the next location (Test, Trigger, and Tape) until all locations were tested and the most painful trunk movement (reference motion) could be performed with an acceptable pain level needed for training (PNRS ≤ 2).

**Train:** The exercises were performed in the directional preference (obtained from the baseline test). The directional preference training programs adopted from McKenzie therapy are grounded in current evidence for managing LBP [20]. A systematic review found that training in the preferred direction can lead to improvements in pain and disability in patients with LBP [50]. The same exercise program as in the EGR was performed only in this case in the directional preference. The exercises consisted of (a) Flexion direction program: recumbent bike, abdominal crunch machine, chess press machine, child pose Pilates position, and Pilates half rollback; or (b) Extension direction program: crosstrainer, lower back extension machine, lat pulldown, puppy Pilates position, and Pilates-breaststroke exercise (Table S2, Training Program).

### 2.9. Sample Size

The sample size analysis was calculated for the Repeated Measure ANOVA-mixed design with the Gpower^©^ program. Based on our a priori results [32], the power analysis was carried out using the following values: Power 0.80, Alfa 0.50, and Sample size effect f^2^ = 0.18 resulting in a minimum total sample size of 46 individuals in a repeated-ANOVA. Ten percent was added to these 46 individuals (*n* = 51), to anticipate possible dropout, which is in line with the COSMIN [51].

### 2.10. Statistical Analysis

Statistical analysis was performed using SPSS^®^ (version 29.0). The outcome variables obtained from the measurements were used to calculate the sample mean, standard deviation, and confidence interval.

Statistical analysis was conducted on data from a total of 51 individuals. Individuals were analyzed according to their initial group allocation. The Kolmogorov–Smirnov test was used to determine the normality of data distribution. Subsequently, the Student’s *t*-test, and the Mann–Whitney U test were used to determine the homogeneity of the baseline variance (Table 1).

A mixed repeated measure ANOVA test was utilized to study the effects on the quality of life, PNRS, and sagittal ROMs. The change in time was analyzed within Baseline-W3-W6-W12 and the difference between 4xT-group and EGR. Before the mixed repeated measure ANOVA was interpreted, Mauchly’s test of sphericity was evaluated; an epsilon adjustment (Greenhouse Keiser at ε < 0.75, Huynh–Feldt at ε > 0.75) was used when lack of sphericity was present. The magnitudes of the treatment effect size within time were considered in calculating the partial eta squared (η_p_^2^) [52]. A η_p_^2^ between 0.01–0.06 was considered as a low, a η_p_^2^ between 0.06–0.014 as a moderate, and above 0.14 as a large effect [53,54]. A statistically significant effect was accepted when a *p*-value < α = 0.05 was achieved. Analyses were performed according to an intention-to-treat principle.

## 3. Results

**Figure 2:**
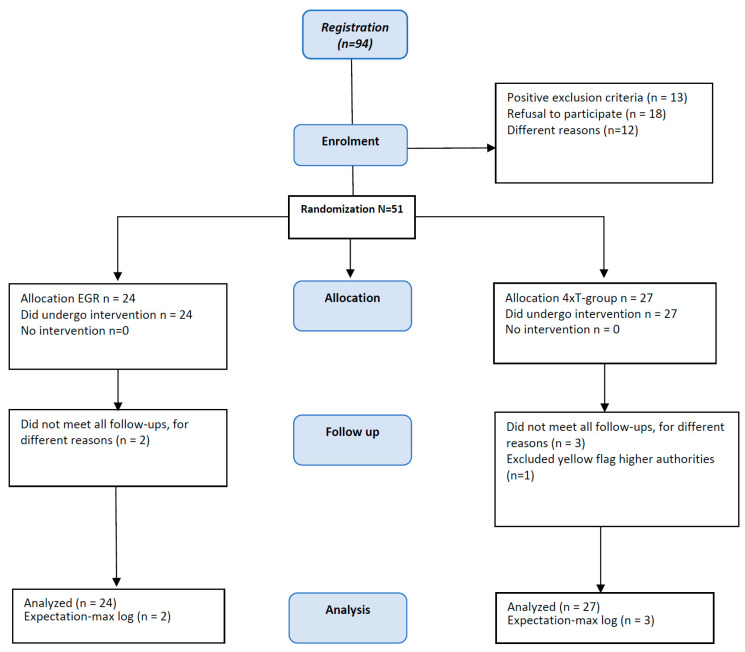
CONSORT diagram of the study design.

In total, 94 individuals were registered for participation in this study. Thirteen individuals were directly excluded during telephone screening. Eighteen individuals decided to retreat from participation after receiving study information. Twelve individuals were eliminated because they met the exclusion criteria. Fifty-one individuals were enrolled in this study. Five individuals did not meet the follow-up (W12) for different reasons (*n* = 2, 4xT; *n* = 3, EGR). The fifty-one individuals were randomized to either the EGR (*n* = 24) or 4xT-group (*n* = 27) (Figure 2). A total of 46 individuals completed the follow-up (90%). For the five dropout cases during the follow-up, the expectation-maximization to manage missing data was performed. The groups were similar at baseline, at that moment, none of the outcome measures were significantly different (Table 1). The variables within each group had a normal distribution (*p* < 0.05).

### 3.1. Level of Pain

**Figure 3:**
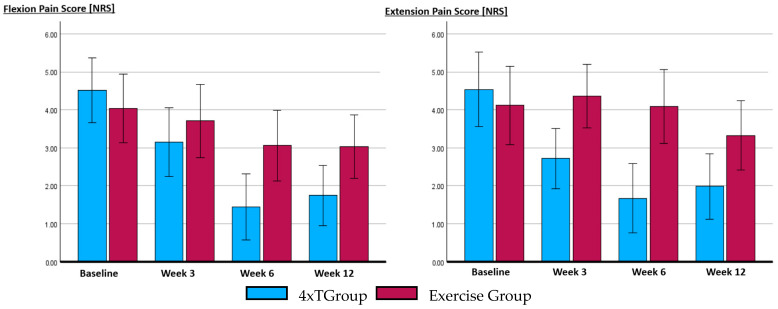
Comparison between the 4xT group and the Exercise group (including sham tape) regarding the outcomes at different measurement time points. The level of pain was rated for the end flexion and end extension motion without elastic tape in situ. All tests and measures were conducted at various time points: before allocation to the group (Baseline), during the 3rd week of the treatment period (Week 3), after the 6-week therapy-on period (Week 6), and following a 6-week therapy-off period (week 12). Abbreviation: NRS, numerical rating scale (0–10).

The level of pain measured with the PNRS showed a significant interaction between times and groups for both PNRS measurements during flexion (*p* < 0.023, η_p_^2^ = 0.18) and extension (*p* < 0.006, η_p_^2^ = 0.23), indicating a substantial impact. Sphericity was violated in the EGR group (Mauchly’s W = 0.553, *p* < 0.03, ε < 0.75) and not in the 4xT group for PNRS_F_. For PNRS_E_, sphericity was violated in both the 4xT group (Mauchly’s W = 0.571, *p* < 0.02, ε > 0.75) and EGR group (Mauchly’s W = 0.535, *p* < 0.02, ε < 0.75). The level of pain did not change in the EGR group; however, it changed significantly in the 4xT group for both flexion (*p* < 0.001, η_p_^2^ = 0.69) and extension (*p* < 0.001, η_p_^2^ = 0.38). The Bonferroni post hoc test revealed that both PNRS_F_ and PNRS_E_ decreased significantly from baseline up to W6 (*p* < 0.001), but not between W6 and W12 (Figure 3).

### 3.2. Trunk Range of Motion

The reliability of the trunk ROM was ensured by estimating the intra-observer agreement between the operator and blinded assessor during all measure times ((flexion, ICC = 0.98, CI95% 0.95–0.99) and (extension, ICC = 0.97, CI95% 0.93–0.99)).

The trunk mobility showed a significant interaction between times and groups for both ROM measurements during flexion (*p* < 0.005, η_p_^2^ = 0.24) and extension (*p* < 0.004, η_p_^2^ = 0.25), indicating a substantial impact. The assumption of sphericity for FROM was violated in the 4xT group (Mauchly’s W = 0.521, *p* < 0.006, ε < 0.75) and EROM (Mauchly’s W = 0.302, *p* < 0.001, ε < 0.75). Sphericity was not violated in the EGR group.

**Figure 4:**
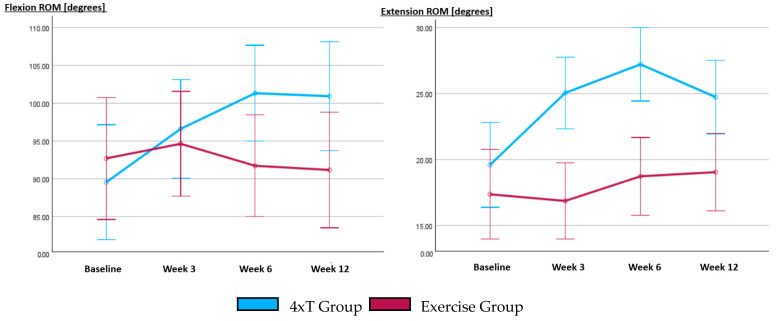
Comparison between the 4xT group and the Exercise group (including sham tape) regarding the outcomes at different measurement time points. The trunk range of motion was measured for the flexion and extension motion without elastic tape in situ. All tests and measures were conducted at various time points: before allocation to the group (Baseline), during the 3rd week of the treatment period (Week 3), after the 6-week therapy-on period (Week 6), and following a 6-week therapy-off period (week 12).

No significant changes were found for LROM in both groups; in addition, no significant changes were found in FROM and EROM in the EGR. The data showed a significant change in 4xT group for FROM (*p* < 0.001, η_p_^2^ = 0.28) and EROM (*p* < 0.001, η_p_^2^ = 0.31). The Bonferroni post hoc test revealed that the FROM and EROM increased significantly from baseline up to W6 (*p* < 0.05). There was no significant relapse found after the therapy-off period for FROM (W6-W12), though it relapsed for EROM after the therapy-off period (W6-W12) (Figure 4).

### 3.3. Quality of Life

The interpretation of the quality of life, as measured by the EQ-5D-3L, was hindered by a significant interaction observed between the factors of time and groups (*p* < 0.002, η_p_^2^ = 0.26). For EQ-5Dl-3L, sphericity was violated in both the 4xT group (Mauchly’s W = 0.459, *p* < 0.002, ε < 0.75) and EGR group (Mauchly’s W = 0.581, *p* < 0.04, ε < 0.75).

The EQ-5D-3L did not change in the EGR group; however, it changed significantly in the 4xT group (*p* < 0.001, η_p_^2^ = 0.47).

The Bonferroni post hoc test revealed that the quality of life measured with the EQ-5D-3L increased significantly from baseline up to W12 (see Table 2). The quality of life measured with the EQ-VAS showed a significant improvement within time for both groups (*p* < 0.001). There was no significant difference between the groups on EQ-VAS score.

## 4. Discussion

The current study found that the 4xT method in NSCLBP individuals was effective in reducing mobility-dependent pain, increasing trunk ROM, and improving quality of life in comparison to exercise therapy only, including paravertebral sham elastic tape.

The PNRS, EROM, and FROM improved significantly in the 4xT-group and not in the EGR. The FROM improved at all time points in the 4xT-group, though, not for LROM. The EROM and FROM in this study were measured by placing the inclinometer at the height L1/T12, which measures the total ROM and is not able to distinguish hip from trunk mobility [37]. Hence, it cannot be stated that the change in mobility was explained by increased trunk ROM. The PNRS was rated at the end of the trunk motion, which may influence an individual’s mobility. The literature consistently highlights a reciprocal relationship between trunk mobility and pain [55,56]. Evaluating pain levels at the end of trunk motion may indicate how pain impacts an individual’s movement capabilities. In the context of NSCLBP, patients reveal a significant inverse correlation between their quality of life, the severity of LBP [57], levels of disability [58], and functional limitations [59]. The noteworthy increase in FROM, EROM, and the reduction in PNRS during these trunk motions observed in the 4xT group raises potential interesting interactions. These positive physical outcomes may be associated with a simultaneous increase in EQ-5D-3L score.

Within the scope of managing LBP, both the literature and clinical guidelines in the field of physical therapy propose a variety of interventions, spanning from manual techniques to exercise therapy and health education [16,17,18,19,20]. However, the treatment effects of the suggested interventions, encompassing both manual techniques and exercises, are reported to be low to moderate [60]. Hence, the LBP clinical guidelines are critically discussed at the 11th Interdisciplinary World Congress on Low Back & Pelvic Girdle Pain, Melbourne, Australia, due to their lack of validity [61]. A more individualized approach is a common practice that has the potential to be more effective for people with LBP [21]. This recommendation aligns with the findings reported in investigations of elastic tape, myofascial release, or spinal joint mobilization when combined with exercise [62,63,64,65,66]. In addition, the American Physical Therapy Association has issued guidelines that suggest using preferred directions for exercise and joint mobilization to treat LBP, emphasizing a more individualized approach [20]. Although these directional preference methods have shown promise in LBP treatment, studies have not demonstrated their superiority in reducing pain, improving function, or decreasing disability compared to other therapeutic approaches [66,67,68,69,70]. To determine the preferred directions of each NSCLBP individual in the 4xT group, the methodology involved displacing the skin during the reference test in accordance with the DAMT-Test [30]. Van Amstel et al. indicated that the DAMT-Test’s SKD instantaneously influences thoracic, lumbar, and hip ROM, with the change in ROM being both direction- and location-dependent [29]. These effects demonstrated significant statistical outcomes and effect sizes, along with good intra- and inter-tester reliability [29]. According to a case study, during the initial treatment, the 4xT was found to be effective in reducing pain and increasing trunk mobility when the interventions were performed in the direction obtained from the DAMT-Test [31]. Although in this study, the change in pain, trunk mobility, and quality of life was measured at Baseline-W3-W6-and W12, it is unclear how each individual responded to each step (Test, Trigger, and Tape) and the result of one treatment session on pain and mobility is not reported. The report also does not indicate whether each individual was able to train with an acceptable pain level. Additionally, the follow-up results were evaluated after a short 6-week therapy-off period, and the longer-term results are unknown.

The spinal joint mobilizations in the 4xT-group were performed in a standing position; the feasibility and mobilizing effect of lumbar mobilization in a full standing position require more information. Moreover, information on how FTMs can influence trunk mobility does need more insight. Mathematical geometric modeling shows that forces applied to the skin can deform and displace the underlying fasciae and as such change the mechanical properties of the underlying fasciae and muscles [71,72]. However, the working mechanism lacks and is based on theoretical models. It is unclear whether the trunk mobility in the 4xT-group is changed due to changes in elasticity (Young’s modulus) of the tissues encompassing and comprising the spine and/or by changes in neuromuscular reflexes. In the 4xT-group, the FTMs are applied in the SKD direction with a positive effect on pain and ROM. An explanation for the SKD effect on pain and joint mobility might be that the relative position between the superficial fascia, thoracolumbar fascia, superficial back muscles, and deep back muscles changes the neuromuscular reflexes during trunk motion. Research reveals that displacing the lumbodorsal skin during trunk motion and using tape consecutively impacts spine and hip mobility [29,73]. Animal studies show that changing the relative position and length of a skeletal muscle via epimuscular tissues provokes a muscular reflex of adjacent skeletal muscles, which is load-direction-dependent [74], and this phenomenon may also be present in humans [29,75]; however, there is a lack of evidence.

In conclusion, more research is needed to investigate the effectiveness of the 4xT in high-quality studies. In addition, further research is required to validate the practical utility of the DAMT-Test and its ability to predict the effects of myofascial release, elastic tape, and joint mobilization on changes in pain and joint ROM.

## 5. Strengths and Limitations

A strength of the study is the robust internal validity of the study, because the treatment consisted of a standardized treatment protocol that was performed by a trained physiotherapist [30], a reliable standardized clinical measure method was used [32,37], there was a sufficient sample size (*n* = 51), the use of Mauchly’s test of sphericity to evaluate statistical significance, and the comparison of treatment effects with exercise, including sham elastic tape.

The limitations of this study include the small sample sizes used to assess the within effects, the absence of investigation into the presence of central sensitization, the use of the expectation-maximization to manage missing data, and the utilization of mobility and pain measurement instruments with low psychometric quality, which may have affected the accuracy and reliability of the study’s findings.

## 6. Implications

This study investigated the effects of the 4xT method on clinical outcomes in individuals with NSCLBP. Participants underwent two weekly treatments for six weeks, each incorporating various FTMs with different mechanical effects. This implies that the FTMs used in the 4xT approach may have varied impacts on the overall body system. Furthermore, biomechanical effects can be classified into instantaneous, acute, mid-term, and long-term effects, considering the treatment frequency and period. Currently, the fundamental biomechanical understanding of FTMs relies on indirect acute post-intervention effects and, more recently, instantaneous effects. However, the mid and long-term effects of FTMs are primarily based on theoretical models. The subsequent section will delve into the potential underlying mechanisms of FTMs used in the 4xT method, forming the rationale for our study.

### The Underlying Mechanism of Fascia Tissue Manipulations

In a healthy condition, when flexing the spine, the skin, superficial fascia, and thoracolumbar fascia are expected to be strained and subjected to shear stress, causing them to slide over the deep back muscles [76,77]. Individuals with NSCLBP exhibit uniform thickening of the thoracolumbar fascia, contrasting with healthy controls where thickness demonstrates variability between the left and right sides, expected to have different anisotropic behavior during spinal motion [78].

During passive lumbar flexion in individuals with LBP, the sliding mobility of the thoracolumbar fascia over the erector spinae muscle is substantially reduced (≈20%) compared to healthy controls [76]. The reduced sliding mobility is associated with deviations in erector spinae muscle activity [79], flexion mobility, and extension mobility [76]. It is speculated to be the cause of changes in mechanical properties of the tissues and adhesions between the thoracolumbar fascia and erector spinae muscle [79,80]. The altered tissue-specific mechanical properties cause a strained condition that is expected to be restored and resolved through FTMs.

The rationale underlying FTMs, is that the skin is an important structure that allows stress transmission onto underlying structures during skin displacements [29,71,72,81]. The generated force during SKD is expected to be transmitted via the lumbodorsal superficial fascia to the thoracolumbar fascia, myofascia, muscles, and thoracic, lumbar, and hip arthrofascia, since they are linked via connective tissues [82,83,84,85]. These forces stress and strain the aforementioned structures, and may influence the interfascial and fasciae–muscle relative positions [86]. Lumbodorsal myofascial releases, stress and straining fascia, and muscle tissues alleviate inflammatory responses and target extracellular protein deposition [87]. This has been shown to lead to altered thoracolumbar fascia thickness and elasticity [88,89], thoracolumbar fascia stress–strain ratios, and latissimus dorsi force output [90], improved transverse abdominal sliding mobility [27], and a reduction in erector spinae myoelectric activity during trunk flexion in NCLBP individuals [75]. Spinal mobilizations and high-velocity thrust manipulations can be considered FTMs when the aim is to reduce arthrofascial and myofascial stiffness [81]. Spinal mobilizations and high-velocity thrust manipulations have been shown to result in changes in muscle activity, increased mobility, decreased pain, and modifications in spinal tissue behavior [91]. Elastic tape is described to align and provide tension to the fascia underneath the elastic tape [49]. The application of paraspinal elastic tape was found to increase the stiffness of the lumbodorsal fascia and the paraspinal muscles beneath the tape [82,92]. Research involving rats suggests that changing the length of the myofascial unit has an impact on reflexes in nearby skeletal muscles [74]. This tensile stress activates muscle spindles [93], which may increase or reduce muscle involuntary contraction [94]. Based on these studies, it is hypothesized that in individuals with LBP, SKD can instantaneously destress or stress the strained tissue due to local tissue deformation and changing the relative position between tissues (muscles and fasciae). This modulation of sensorimotor interaction may influence muscle activity, pain perception, and joint range of motion [29], providing a potential explanation for the observed instantaneous effects of SKD on joint mobility in healthy individuals [29].

These sources reveal that myofascial releases potentially reduce tissue stiffness, while elastic taping, in contrast, increases tissue stiffness, indicating the potential for optimizing local stiffness essential for free movement. However, achieving the appropriate stiffness requires individualization. Clinicians should strategically insert optimal stiffness in one direction at a specific location and, conversely, insert optimal flexibility in another direction at a different location to fine-tune a linkage within the fascial system. Determining the most appropriate stiffness at specific locations and in specific directions poses a challenge. The DAMT-Test [30] may serve as a valuable clinical indicator of the need for FTMs [29] by altering the tissue stiffness by SKD during joint motion to assess the impact on pain and mobility at a specific location and direction [31].

This may support the effectiveness of the results found in this study. However, it is crucial to approach the application of these findings to broader clinical practice with careful consideration. Further research is necessary to comprehend the underlying mechanism and evaluate the predictive power of the DAMT-Test in determining the effects of FTMs on changes in mobility and pain, as used in the 4xT method for addressing musculoskeletal disorders.

## 7. Conclusions

The results of this study demonstrate that the 4xT method stands out as a promising and impactful treatment option for individuals dealing with nonspecific chronic low back pain. The substantial reductions in pain, increase in trunk mobility, and the notable improvement in overall quality of life, when contrasted with exercise-only therapy, emphasize its potential clinical significance. Specifically, analyzing the application location and direction of fascia tissue manipulations and the direction-specific training guided by the DAMT-Test within the 4xT Back protocol demonstrated lasting effectiveness, evident even after the 6-week therapy-off period. Continued investigation into the application of the 4xT method is warranted.

## Figures and Tables

**Table 1:** Demographic and clinical characteristics of participants at baseline.

	4xT Group	Exercise Therapy Group	
	Mean ± SD	Mean ± SD	*p* Value
Age	44.8 ± SD 10.4	45.0 ± SD 10.0	0.5 ^a^
EQ 5D 3L index	0.49 ± SD 0.30	0.61 ± SD 0.24	0.3 ^b^
EQ-VAS	56 ± SD 20	55 ± SD 16	0.2 ^a^
FROM	89.5 ± SD 20.5	92.7 ± SD 18.7	0.7 ^a^
EROM	19.6 ± SD 9.0	17.3 ± SD 7.4	0.1 ^a^
Lumbar FROM	37.4 ± SD 13.8	36.4 ± SD 12.8	0.1 ^a^
PNRS_F_ (0–10)	4.5 ± SD 2.4	4.0 ± SD 2.1	0.7 ^a^
PNRS_E_ (0–10)	4.5 ± SD 2.6	4.1 ± SD 2.5	0.6 ^a^
Sex	Men *n* = 17	Men *n* = 12	
	Female *n* = 10	Female *n* = 12	
	Total *n* = 27	Total *n* = 24	

This table presents baseline values for the most crucial prognostic indicators. Data is expressed as Mean ± SD, and *p*-values are provided. Abbreviations: SD, standard deviation; ±, plus-minus; ^a^, Levene’s *p*-value of independent sample *t*-test; ^b^, Mann–Whitney; FROM, flexion range of motion; EROM, extension range of motion; PNRS_F_, pain numeric rating score for end flexion; PNRS_E_, pain numeric rating score for end extension; VAS, visual analogue scale; *n*, number of subjects.

**Table 2:** Quality of life EQ-5D-3L-index results.

	4xT Group (*n* = 27)			Exercise Therapy Group (*n* = 24)		
Score per Week	Mean ± SD	CI95%		Mean ± SD	CI95%	
*Baseline*	0.49 ± SD 0.30	−0.11	0.85	0.61 ± SD 0.24	0.03	0.89
*Week 3*	0.72 ± SD 0.24	0.11	1.0	0.59 ± SD 0.24	0.03	0.90
*Week 6*	0.80 ± SD 0.16	0.31	1.0	0.68 ± SD 0.19	0.03	1.0
*Week 12*	0.77 ± SD 0.29	−0.08	1.0	0.68 ± SD 0.31	0.03	1.0
*p* value	<0.001			NS		

All assessments were conducted at various time points: before group allocation (Baseline), during the 3rd week of the treatment period (Week 3), after the 6-week therapy-on period (Week 6), and following a 6-week therapy-off period (Week 12). Quality of life, as measured by the EQ-5D-3L index (ranging from 0 for the worst quality of life to 1.0 for the best quality of life), is presented as Mean ± SD. *p*-values were obtained from the Repeated Measures ANOVA mixed design. Abbreviations: SD, standard deviation; ±, plus-minus; 95% CI, 95 percent confidence interval; NS, not significant; *n*, number of subjects.

## Data Availability

On request, the corresponding author will provide access to data used in this work. Due to ethical constraints, data are not publicly accessible.

## Supplementary Materials

The following supporting information can be downloaded at: https://www.mdpi.com/article/10.3390/life14010007/s1, Video S1: Dynamic ArthroMyoFascial Translation Test Procedure; Table S2: Training program Train 4xT and EGR.
