# Supplementary material for: Fascia Tissue Manipulations in Chronic Low Back Pain: A Pragmatic Comparative Randomized Clinical Trial of the 4xT Method® and Exercise Therapy
Source: Life (Basel). 2023 Dec 20;14(1):7. doi: 10.3390/life14010007 (PMC10820544; doi:10.3390/life14010007)
Supplement: Supplementary file 1 [file life-14-00007-s001.zip › Table S2, Training Program.pdf]

Fascia Tissue Manipulations in Chronic Low Back Pain: A Comparative Randomized Clinical Trial of the 4xT Method® and Exercise Therapy

Robbert van Amstel, Karl Noten, Shaun Malone, and Peter Vaes

Supplement: Table S2, Training Program

Training program

Both groups followed a training program. In the 4xT-group the training was performed in the positive movement, direction-specific (flexion or extension), determined by the Dynamic Arthro-Myofascial Translation Test®, according to the 4xT method [1,2]. This was not the case in the exercise-only group where the subjects performed all exercises of the described training program utilized in this study. In both groups, the training was performed under the supervision of the physiotherapist.

It has to be noticed that no training guideline recommends the proper frequency, intensity, or repetitions for the chosen exercise [3]. The rationale behind the training program is to combine flexibility and resistance exercises, as recommended in the LBP guidelines [4,5]. Flexibility training aims to increase functional mobility [6], while resistance training specifically focuses on promoting muscle growth [7] associated with anti-inflammatory responses [8]. The training protocol focuses on isolating the lumbar multifidus and transverse abdominal muscles during muscle strength training, which are considered core muscles and are essential for trunk stability in all directions [9]. To activate the core muscles during Pilates and machine exercises, anisometric–isotonic and anisometric–anisotonic contractions were performed by the patients, respectively. For muscle endurance resistance training, the patients performed 4 sets of 15 to 20 repetitions at a super-slow tempo (8 s for the concentric phase and 8 s for the eccentric phase) at an intensity of 50% 1RM $\leq$ 1RM. For muscle strength resistance training, the patients performed 4 sets of 8 to 12 repetitions with an eccentric emphasis at an intensity of 70% to 85% of 1RM (1 s for the concentric phase and 4 s for the eccentric phase) [7]. A rest period of 60 s was taken between sets, consistent with the recommendation by Schoenfeld et al. [7].

The safety of the exercises where ensured given instructions that met the criteria for the exercises [10]. To exclude inappropriate execution the participants were supervised 2 times a week by their physiotherapists. These exercises were as follows in sequence performed (2 times a week):

**Training Program.** Exercise-only group: flexion and extension direction			
**Exercise**	**Intensity**	**Repetition**	**Core contraction**
Recumbent bike	3-6 METS	10 minutes	anisometric – anisotonic
Abdominal crunch machine	30-50% 1RM	20-20-15	anisometric – isotonic
Chess press machine	60-85% 1RM	15-12-8	anisometric – anisotonic
Half rollback (pilates)	-	15-12-10	anisometric – isotonic
Child pose (pilates)	-	2 x 1 minute	anisometric – isotonic
Back extension machine	30-50% 1RM	20-20-15	anisometric – isotonic
Lat pull down	60-85% 1RM	15-12-8	anisometric – anisotonic
Breaststroke (pilates)	-	15-12-10	anisometric – isotonic
Low or high puppy (pilates)	-	2 x 1 minute	-
Crosstrainer	3-6 METS	5 minutes	anisometric – anisotonic
Abbreviation: METs, Metabolic Equivalent of Task; 1RM, one Repetition Maximum;<br>The training is supervised by a trained physiotherapist. Between each repetition, 60 seconds of rest was held.<br>During the training, the weights and repetitions were recorded in a logbook.			

**Training Program.** 4xT- group: flexion or extension direction				
**Exercise**	**Intensity**	**Repetition**	**Core contraction**	**Direction**
Recumbent bike	3-6 METS	10 minutes	anisometric – anisotonic	Flexion
Abdominal crunch machine	30-50% 1RM	20-20-15	anisometric – isotonic	
Chess press machine	60-85% 1RM	15-12-8	anisometric – anisotonic	
Half rollback (pilates)	-	15-12-10	anisometric – isotonic	
Child pose (pilates)	-	2 x 1 minute	-	
Recumbent bike	3-6 METS	5 minutes	anisometric – anisotonic	
Bike (Upright)*	3-6 METS	10 minutes	anisometric – anisotonic	Extension
Back extension machine	30-50% 1RM	20-20-15	anisometric – isotonic	
Lat pull down	60-85% 1RM	15-12-8	anisometric – anisotonic	
Breaststroke (pilates)	-	15-12-10	anisometric – isotonic	
Low or high puppy (pilates)	-	2 x 1 minute	-	
Crosstrainer	3-6 METS	5 minutes	anisometric – anisotonic	

Abbreviation: METs, Metabolic Equivalent of Task; 1RM, one Repetition Maximum;

The training is supervised by a trained physiotherapist. Between each repetition, 60 seconds of rest was held. During the training, the weights and repetitions were recorded in a logbook. * Patient was seated in an upright position on the bike and was instructed to avoid slouching posture.

References.

1. Noten, K. The Dynamic ArthroMyofascial Translation® Test (DAMT®Test): 2012. (4xT®Method the ArthroMyofascial Therapy: Chapter 2). **2021**, 10-15, doi:https://doi.org/10.17605/OSF.IO/D85K3.
2. Noten, K. *MedicalFitness RUGKLACHTEN. De beste therapeutische training*, 1 ed.; Fysio Physics Media & Publishing: 2022.
3. Staal, J.B.; Hendriks, E.; Heijmans, M.; Kiers, H.; Lutgers-Boomsma, A.M.; Rutten, G.; van Tulder, M.W.; den Boer, J.; Ostelo, R.; Custer, J.W.H. KNGF Clinical Practice Guideline for Physical Therapy in patients with low back pain. *KNGF-richtlijn* **2017**.
4. Swart, N.M.; Apeldoorn, A.T.; Conijn, D.; Meerhoff, G.A.; Ostelo, R. KNGF Clinical Practice Guideline for Low back pain and lumbosacral radicular syndrome. *KNGF-richtlijn* **2021**.
5. Zambelli, Z.; Halstead, E.J.; Iles, R.; Fidalgo, A.R.; Dimitriou, D. The 2021 NICE guidelines for assessment and management of chronic pain: A cross-sectional study mapping against a sample of 1,000* in the community. *British Journal of Pain* **2022**, *16*, 439-449.
6. Yu, Z.; Yin, Y.; Wang, J.; Zhang, X.; Cai, H.; Peng, F. Efficacy of Pilates on Pain, Functional Disorders and Quality of Life in Patients with Chronic Low Back Pain: A Systematic Review and Meta-Analysis. *International Journal of Environmental Research and Public Health* **2023**, *20*, 2850.
7. Schoenfeld, B.J.; Peterson, M.D.; Ogborn, D.; Contreras, B.; Sonmez, G.T. Effects of low-vs. high-load resistance training on muscle strength and hypertrophy in well-trained men. *The Journal of Strength & Conditioning Research* **2015**, *29*, 2954-2963.
8. Jiang, Q.; Lou, K.; Hou, L.; Lu, Y.; Sun, L.; Tan, S.C.; Low, T.Y.; Kord-Varkaneh, H.; Pang, S. The effect of resistance training on serum insulin-like growth factor 1 (IGF-1): a systematic review and meta-analysis. *Complementary therapies in medicine* **2020**, *50*, 102360.
9. Frizziero, A.; Pellizzon, G.; Vittadini, F.; Bigliardi, D.; Costantino, C. Efficacy of core stability in non-specific chronic low back pain. *Journal of functional morphology and kinesiology* **2021**, *6*, 37.
10. Monfort-Pañego, M.; Vera-García, F.J.; Sánchez-Zuriaga, D.; Sarti-Martínez, M.Á. Electromyographic studies in abdominal exercises: a literature synthesis. *Journal of manipulative and physiological therapeutics* **2009**, *32*, 232-244.
